# A Case of Loss of Speech Induced by Compression on the Brain, Which Continued for Some Months

**Published:** 1823-09

**Authors:** Michael Ryan

**Affiliations:** Member of the Royal College of Surgeons, London; and Licentiate of the Royal College of Surgeons, Edinburgh, &c. &c.


Art. VII.—*A Case of Loss of Speech induced by Compression on the Brain, which continued for some Months.* By Michael Ryan, M.D. Edin.; Member of the Royal College of Surgeons, London; and Licentiate of the Royal College of Surgeons, Edinburgh, &c. &c.

J. D. aged fourteen, a stout healty boy, a labourer, received a blow on the head, which caused a depressed fracture, about one inch in circumference, on the anterior inferior angle of the left parietal bone, which was immediately followed by well-marked symptoms of compression on the brain. I was requested to see him about thirteen hours after he received the injury, and found him labouring under stupor, stertorous breathing, pupils dilated and motionless, with the pulse slow and very full. Venesection and arteriotomy were largely employed; alvine evacuations with difficulty procured; cold lotions and a blister were applied to the head; and the antiphlogistic regimen most strictly adhered to: all with beneficial effects, as the urgent symptoms were removed, except paralysis of the tongue and loss of speech. This treatment had been repeated according to the urgency of symptoms; he gradually regained the use of all his faculties, except that of speech. Rubifacient applications and sinapisms were frequently applied to the external fauces and nape of the neck, as also topical bleeding and blistering, without any good effects. Digitalis was exhibited, with a view of increasing absorption of the effused fluids in the brain; an effect which I had witnessed, on a similar occasion, to result from the use of that medicine.

About three months from the date of the injury, he could articulate the monosyllable No; his tongue was free from paralysis, and his hearing was wonderfully acute. He continued speechless, however, for a further period of five months; during which the local applications were occasionally employed, together with an issue in the neck, without any apparent advantage. Yet he gradually regained the use of the lost faculty, his articulation resembling that of a person recovering from apoplexy, but daily becoming more perfect.

It is now ten months since I saw him, and for the last three of which he has abandoned the use of medicine altogether. The motion of the lips, cheeks, tongue, and power of deglutition, as well as the senses of vision and hearing, became natural, while speech was absent; and the patient frequently shed tears when spoken to, being unable to reply to the interrogations of those who addressed him. The tongue had been paralysed for several days after he regained the power of deglutition; and, when his voice returned, he made daily efforts to articulate before he succeeded.

Speech may be injured or suppressed by diseases of the nerves which supply the organs that assist in producing that noble faculty,—by pressure or wounds on those cerebral productions; by affections of the larynx, its muscles, ligaments, cartilages; by disorders of the glottis, trachea, fauces, palate, sinuses; by tumors or polypi; by ozena, cancer of the lips, cheeks, or nose; by inflammation, enlargement, schirrous cancer, or congenital vitiation of the tongue; or by its frænum being too short. Forestus* describes inflammation and suppuration of

* *Observ.* l. 24, c. 14.

that organ; and Galen mentions a case of œdematous tumor of it, so great as to cause it to be protruded from the mouth;* and a second case,† which did not proceed from schirrus or œdema, though it increased to a considerable extent, but from too copious a supply of aliment deposited in its substance. Claudinus was consulted in the case of a girl, aged twelve, whose tongue was wonderfully enlarged, not from œdema, schirrus, or other vitiation of its structure, as it readily diminished by pressure; but it proceeded from a rupture of the frænum linguæ, which caused a great determination of blood to that organ; and, strange to relate, this tumor was said to increase in the morning and become livid, while it declined in the evening!‡ I have lately seen a case of schirrous enlargement of the tongue, which was greatly aggravated by the use of mercury, and which suppressed the faculty of speech, and ultimately destroyed the patient. I divided the frænum linguæ in a girl, aged ten, who could scarcely speak, yet obtained an evident degree of fluency immediately after the operation. If both recurrent nerves which supply the larynx be divided, speech and voice will be abolished; if a wound be made below the larynx, voice will be suppressed; if above it, speech will be destroyed; and the diminution or destruction of nervous power in the fifth pair of nerves is the principal cause of loss of motion in the tongue. Avicenna relates a case of aphonia, induced by the application of deep scarifications to the occiput, which, he said, wounded some of the nerves that proceed from the cerebellum through that part. If cutaneous eruptions be suddenly repelled, or congestion of blood take place in the fauces or tongue; or the suppression of periodical evacuations in plethoric habits; or the influence of fear or other passions; the too free use of spirituous potations, or whatever injures or destroys the parts connected in producing voice or speech, will cause an abolition of that faculty. Certain spasmodic diseases, as hysteria, also effect it. I have seen two cases, in both of whom the incisores teeth had been displaced by external violence; they laboured under paraphonia until these substances had been replaced, after which the voice and speech became natural, and the teeth perfectly firm. In low fevers, or in apoplexy, the voice is regained slowly; and in the case of a young lady, who was in the last stage of phthisis, I lately observed this clearly illustrated: she lost the power of speech for thirty hours, appeared moribund, pulse scarcely perceptible; the oleum ammoniatum was applied to the external fauces, which caused several feeble motions of the tongue and efforts to speak; which faculty she

* Gal. lib. 14, met. c. 8.
† *Op. cit.* lib. *de Diff. Morb.* c. 9.
‡ Claud. *Consul.* 9.

gradually regained, and retained to the third day, when she expired.

The prognosis in aphonia is mostly unfavourable, and the complaints very slow and tedious as to alleviation. General and local bleeding, cathartics, the antiphlogistic regimen, with other stimulant applications to the occiput, fauces, tongue, masticatories, and errhines, with those other means which symptoms may require, form the best mode of treating this melancholy disease.

*Patrick-street, Kilkenny; July 30th,* 1823.

